# Predicting Fetal Alcohol Spectrum Disorders Using Machine Learning Techniques: Multisite Retrospective Cohort Study

**DOI:** 10.2196/45041

**Published:** 2023-07-18

**Authors:** Sarah Soyeon Oh, Irene Kuang, Hyewon Jeong, Jin-Yeop Song, Boyu Ren, Jong Youn Moon, Eun-Cheol Park, Ichiro Kawachi

**Affiliations:** 1 Department of Social and Behavioral Sciences Harvard TH Chan School of Public Health Boston, MA United States; 2 Institute of Health Services Research Yonsei University College of Medicine Seoul Republic of Korea; 3 Department of Electrical Engineering and Computer Science Massachusetts Institute of Technology Cambridge, MA United States; 4 Department of Physics Massachusetts Institute of Technology Cambridge, MA United States; 5 Department of Psychiatry Harvard Medical School Boston, MA United States; 6 Artificial Intelligence and Big-Data Convergence Center Gil Medical Center Gachon University College of Medicine Incheon Republic of Korea

**Keywords:** fetal alcohol syndrome, machine learning, algorithm, development, fetal, fetus, maternal, obstetric, gynecology, pregnant, prenatal, antenatal, postnatal, predict, developmental disability, prenatal alcohol exposure, alcohol, alcohol exposure, developmental, disability, pregnancy, age, race, diagnosis, diagnostic, treatment

## Abstract

**Background:**

Fetal alcohol syndrome (FAS) is a lifelong developmental disability that occurs among individuals with prenatal alcohol exposure (PAE). With improved prediction models, FAS can be diagnosed or treated early, if not completely prevented.

**Objective:**

In this study, we sought to compare different machine learning algorithms and their FAS predictive performance among women who consumed alcohol during pregnancy. We also aimed to identify which variables (eg, timing of exposure to alcohol during pregnancy and type of alcohol consumed) were most influential in generating an accurate model.

**Methods:**

Data from the collaborative initiative on fetal alcohol spectrum disorders from 2007 to 2017 were used to gather information about 595 women who consumed alcohol during pregnancy at 5 hospital sites around the United States. To obtain information about PAE, questionnaires or in-person interviews, as well as reviews of medical, legal, or social service records were used to gather information about alcohol consumption. Four different machine learning algorithms (logistic regression, XGBoost, light gradient-boosting machine, and CatBoost) were trained to predict the prevalence of FAS at birth, and model performance was measured by analyzing the area under the receiver operating characteristics curve (AUROC). Of the total cases, 80% were randomly selected for training, while 20% remained as test data sets for predicting FAS. Feature importance was also analyzed using Shapley values for the best-performing algorithm.

**Results:**

Overall, there were 20 cases of FAS within a total population of 595 individuals with PAE. Most of the drinking occurred in the first trimester only (n=491) or throughout all 3 trimesters (n=95); however, there were also reports of drinking in the first and second trimesters only (n=8), and 1 case of drinking in the third trimester only (n=1). The CatBoost method delivered the best performance in terms of AUROC (0.92) and area under the precision-recall curve (AUPRC 0.51), followed by the logistic regression method (AUROC 0.90; AUPRC 0.59), the light gradient-boosting machine (AUROC 0.89; AUPRC 0.52), and XGBoost (AUROC 0.86; AURPC 0.45). Shapley values in the CatBoost model revealed that 12 variables were considered important in FAS prediction, with drinking throughout all 3 trimesters of pregnancy, maternal age, race, and type of alcoholic beverage consumed (eg, beer, wine, or liquor) scoring highly in overall feature importance. For most predictive measures, the best performance was obtained by the CatBoost algorithm, with an AUROC of 0.92, precision of 0.50, specificity of 0.29, F1 score of 0.29, and accuracy of 0.96.

**Conclusions:**

Machine learning algorithms were able to identify FAS risk with a prediction performance higher than that of previous models among pregnant drinkers. For small training sets, which are common with FAS, boosting mechanisms like CatBoost may help alleviate certain problems associated with data imbalances and difficulties in optimization or generalization.

## Introduction

Fetal alcohol spectrum disorders (FASDs) comprise a range of neuropsychological and behavioral deficits that emerge from prenatal alcohol exposure (PAE) [1]. The most severe form of FASD, otherwise known as fetal alcohol syndrome (FAS), is characterized by distinct facial malformations, prenatal or postnatal growth retardation, and central nervous system abnormalities [2]. In the United States, it is predicted that around 1%-5% of school-aged children have FASDs and 0.6%-0.9% have FAS [3]. While FASDs are 100% preventable if a pregnant woman abstains from consuming alcohol [4], more than 10% of women drink during pregnancy [5]. For certain populations, such as women with alcohol use disorders (AUDs) and women with unintended pregnancies, this rate is higher [6].

Machine learning (ML) proposes an interesting solution to predicting FASDs, as only 1 in every 13 pregnant women who consume alcohol during pregnancy delivers a child with FASDs [7]. However, studies using ML algorithms in FAS detection have been limited to small samples [8] or nonhuman studies [9]. For example, a rodent study of FAS found that certain ML models, including support vector machine–based algorithms of the brain’s functional connectivity, have successfully predicted PAE among rodents with an accuracy of up to 62.5%, highlighting the potential for ML-based human subject research [9]. Genome-wide DNA methylation data in small human cohorts (n=48) have achieved moderately accurate predictions of FASD status by using gradient boosting models to distinguish FASD cases and controls [8]. The “K Nearest Neighbor” algorithm for imputing missing prenatal alcohol data has predicted pregnant drinkers with an accuracy of up to 76% and shown potential in imputing missing data for longitudinal studies where data missingness leads to bias [10].

As of now, few studies have attempted to use ML strategies to detect prenatal exposure to alcohol, despite its increased use in retrospective studies of other teratogens like tobacco [11,12], environmental contaminants [13], and certain medications [14]. For example, in an American cohort study of 531 children between 3 and 5 years old, ML algorithms achieved an accuracy of 81% in detecting prenatal exposure to smoking, by incorporating DNA methylation data and maternal self-reports [12]. In another study of longitudinal birth cohorts in New York City, cord blood DNA methylation samples were found to predict average prenatal exposure to air pollution like NO_2_ and PM_2.5_ with an accuracy of up to 60% (95% CI 0.52-0.68) [15].

Furthermore, while ML has been used increasingly in recent years to improve the “diagnosis” of FAS—for example, identifying facial features [16]—fewer studies have focused on “predicting” FAS based upon maternal risk factors such as the timing of alcohol exposure during pregnancy (eg, first vs second or third trimester), as well as the frequency and amount of drinking. One reason is because of the difficulty of collecting detailed information about alcohol drinking during pregnancy [9]. With the creation of the collaborative initiative on fetal alcohol spectrum disorders (CIFASDs) in 2003, a consortium of clinicians and researchers from multiple sites in the United States and Europe have begun to collaboratively gather data on prenatal exposure to drugs and alcohol, including data on alcohol exposure histories from maternal reports and review of medical or legal or social service records [17].

As statistical and ML forecasting methods often vary in predictive performance for neonatal studies (eg, ML methods had higher predictive accuracy than traditional statistical methods in predicting mortality among low birthweight infants) [18], this study aims to predict FASDs based on a number of maternal characteristics, and compare or contrast these factors with risk factors highlighted in the existing body of literature where more traditional, statistical methods were used.

## Methods

### Overview

Data were collected by CIFASD as part of a longitudinal, multisite research study of pregnant drinkers between 2003 and 2017 (for full methodology, see [17]). For this study, data on dysmorphology (U24AA014815), neurobehavior (U01AA014834), and demographics (U01AA014809) were used to gather information about 595 pregnant drinkers who visited 1 of the following sites within the United States to be interviewed about various questions related to their pregnancy behaviors and birth-related outcomes: (1) Center for Behavioral Teratology, San Diego State University, San Diego, CA; (2) Emory University, Atlanta, GA; (3) 7 Northern Plains communities, including 6 Indian reservations; (4) the University of California, Los Angeles, CA; and (5) the University of Minnesota, Minneapolis, MN [17]. Institutional review boards (IRBs) at all CIFASD sites approved this study, and the Harvard T.H. Chan School of Public Health IRB approved analyses of these secondary data (Protocol #: IRB21-1261).

### Study Sites

#### Center for Behavioral Teratology, San Diego, California

At this site, children suspected of alcohol exposure were referred to the principal investigator and local professionals for participation in this project [17]. Many patients were already studying at this center before the initiation of the CIFASD project, including those referred to the investigative team for meeting the traditional diagnostic criteria for FAS (eg, facial anomalies; growth retardation; and evidence of central nervous system dysfunction such as microcephaly, mental retardation, or attentional deficits) [19]. Alcohol exposure histories were obtained via self-reports or professional reviews of medical, legal, or social service records of the biological mother. Parents or primary caregivers completed questionnaires regarding the child’s behavior, while the children were examined for facial features of FAS, that is, 2 of the 3 key facial features (short palpebral fissures: ≤10th centile; thin vermilion border of the upper lip: rank 4 or 5 on a racially normed lip or philtrum guide; smooth philtrum: rank 4 or 5 on a racially normed lip or philtrum guide), as well as signs of prenatal or postnatal growth deficiency (head circumference or height or weight ≤10th percentile) [20].

#### Emory University, Atlanta, Georgia

At Emory University, the Fetal Alcohol and Drug Exposure Clinic gathered data on a large sample of patients with FASDs while providing clinical services and facial evaluations at the Emory University Marcus Institute [21]. In the absence of direct reports, documentation of alcohol abuse or dependence by the biological mother in the form of medical, social services, or court records was reviewed [17]. Recruitment took place via clinical and community referrals, and parents or primary caregivers completed questionnaires or interviews, while patients with FASD were administered various neuropsychological tests over a 3-hour session [22].

#### Northern Plains

Seven communities, including 1 urban and 6 reservation sites throughout North Dakota, South Dakota, and Montana, participated in this study [21]. Children with FASDs were recruited via active case ascertainment methods and advertisements in tribal and community health centers [20]. Data on PAE were obtained from in-person interviews with the parent or primary caregiver to obtain exact exposure histories retrospectively and were also confirmed via reviews of medical records, when available [20].

#### University of Minnesota, Minneapolis, Minnesota

The Department of Psychiatry at the University of Minnesota collected data on PAE histories obtained through several modalities including medical reports, birth records, social service records, and when available, using maternal self-reports [21].

#### University of California, Los Angeles

Data were collected from children attending the Fetal Alcohol and Related Disorders Clinic at University of California, Los Angeles (UCLA) [23]. Participant recruitment was through local FASD clinic referrals, web-based advertisements, and word of mouth in caregiving communities [23]. All alcohol exposure histories were confirmed via in-person interviews, maternal reports of prenatal substance exposure, or the review of maternal medical records by a licensed medical doctor [23].

To obtain information about PAE, questionnaires or in-person interviews as well as reviews of medical, legal, or social service records regarding alcohol-related problems or a diagnosis of alcohol abuse were used to gather information about alcohol consumption. At all CIFASD sites, the Institute of Medicine’s definition of FASDs was used for diagnosis, for example, (1) evidence of a characteristic pattern of minor facial anomalies including at least 2 or more of the key facial features of FAS (palpebral fissures ≤10th centile, thin vermilion border, or smooth philtrum), (2) evidence of prenatal and postnatal growth retardation (height or weight ≤10th centile), (3) evidence of deficient brain growth (structural brain anomalies or occipitofrontal circumference ≤10th centile), and if possible (4), confirmation of maternal alcohol consumption directly from the mother or a knowledgeable collateral source was used for FAS diagnosis [2] were used by dysmorphologists at each site to diagnose FAS. Among children with confirmed PAE, a diagnosis of FAS was made if 2 of the 3 key facial features of FAS (ie, short palpebral fissure, smooth philtrum, or thin vermillion) was accompanied by either microcephaly, growth retardation, or both [17]. Children were excluded when there were reports of known causes of mental deficiency, such as congenital hypothyroidism, neurofibromatosis, or chromosomal abnormalities.

At 4 of the sites including Emory University, the University of California, University of Minnesota, and San Diego, a dysmorphologist trained to accurately diagnose FAS based on physical features, as defined by the CIFASD Dysmorphology Core, was used to diagnose FAS. Contrastingly, for the Northern Plains site, a team of physicians, teachers, and other representatives were trained to identify children with certain morphological characteristics of FASD and other birth defects, IQ, and neuropsychologic traits; however, subjects could not always be referred to a pediatric dysmorphologist for verification or a complete physical examination or morphology assessment; resulting in difficulties with ethnic variations in morphology, syndromic features of FASDs were sometimes compared to normal controls within the same population, in terms of weight, head circumference, fissure length, and other facial characteristics (eg, ptosis and intercanthal distance) [24].

Regarding PAE, all cases of biological mothers with reported alcohol consumption during pregnancy were categorized into 1 of the following mutually exclusive groups: (1) women who consumed alcohol in the first trimester only, (2) women who consumed alcohol in the first and second trimesters only, (3) women who consumed alcohol during all 3 trimesters of pregnancy, and (4) women with “other” drinking patterns (eg, drinking only in the second or third trimester). Preferred alcoholic beverage type (eg, “beer,” “wine,” and “spirits”), maternal age, maternal race (American Indian or Alaska Native, Asian, Native Hawaiian or other Pacific Islander, Black or African American, White, more than 1 race, other), ethnicity (Hispanic or Latino, or Not Hispanic or Latino), the reception of prenatal care (yes, no), and experience of any pregnancy-related complications (bleeding, high blood pressure, diabetes) were also included in all models.

**Figure 1:**
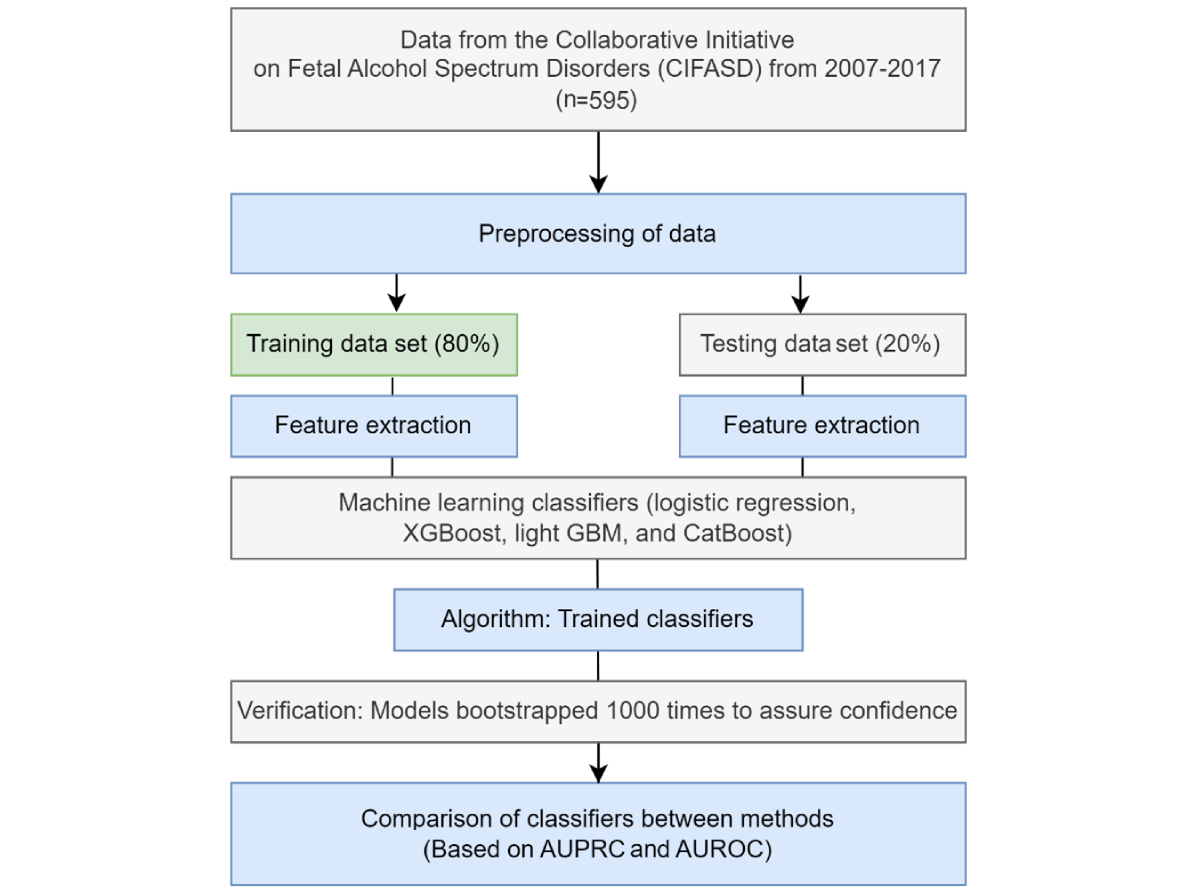
Flow diagram of investigation. AUPRC: area under the precision-recall curve; AUROC: receiver operating characteristics curve; GBM: gradient-boosting machine.

For the computational analyses, 80% (n=595) of the total cases were randomly selected for training, while 20% remained as test data sets for predicting FAS (Figure 1). The measures for the predictive performance of each algorithm (logistic regression, XGBoost, light gradient-boosting machine [GBM], and CatBoost) included area under the precision-recall curve (AUPRC) and area under the receiver operating characteristic curve (AUROC). Besides logistic regression, which has been the traditional approach for making associations between pregnant drinking patterns and FASDs in the existing body of literature [25,26], on boosting (XGBoost [27], light GBM [28], CatBoost [29]), a supervised ML method that consists of aggregating classifiers developed sequentially on the train-test sample, to learn from classifiers, correct errors, and obtain more accurate classifiers by training a sequence of weaker models [30].

In order to verify the prediction performance of our 4 methods, the performance of each of the 4 models was bootstrapped 1000 times for our entire sample (n=595) and validated. A box plot of the mean and pooled standard deviations of each model over 1000 bootstrapped samples can be found in Multimedia Appendix 1. All models had a similar range in variance with pooled standard deviations ranging from 0.07 (light GBM) to 0.09 (CatBoost, XG Boost, logistic regression). As CatBoost continued to show the highest prediction performance out of all 4 models following verification (mean 0.94, SD 0.09), the importance of features based on Shapley values was verified based on the CatBoost model, and Shapley additive explanation values [31] were used to interpret how each feature contributed to the prediction of FAS risk based on the CatBoost model. All statistical analyses were performed using Python (version 3.7.2; Python Software Foundation) and Scikit-learn library (version 0.20.2; David Cournapeau and Matthieu Brucher) [32].

### Ethics Approval

This study was approved by the IRB at the Harvard TH Chan School of Public Health (IRB approval number: IRB21-1261), and the procedures were conducted in accordance with the Helsinki Declaration of 1975, as revised in 2000 for human subjects research. During primary data collection at each clinical location of CIFASD. IRB approval and informed consent were obtained from all adult participants or their legal guardians [33]. For secondary analysis of the data, our research team was provided with deidentified and anonymized data upon request and approval from CIFASD’s data committee.

## Results

Table 1 presents the data characteristics of the study population. Overall, there were 20 cases of FAS within a total population of 595 individuals with PAE. Most of the drinking occurred in the first trimester only (n=491) or throughout all 3 trimesters (n=95); however, there were also reports of drinking in the first and second trimesters only (n=8), and 1 case of drinking in the third trimester only (n=1).

**Figure 2:**
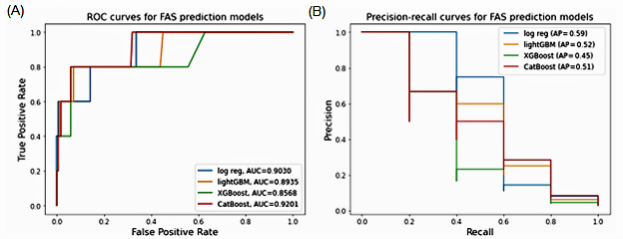
Performance evaluation of machine learning (ML) algorithms for FAS prediction in the collaborative initiative on fetal alcohol spectrum disorder (CIFASD) data set. AUC: area under the receiver operating characteristic curve; AP: average precision; FAS: fetal alcohol syndrome; GBM: gradient-boosting machine; ROC: receiver operating characteristic.

Figure 2 presents the AUROC and the AUPRC of each ML algorithm. The CatBoost method delivered the best performance in terms of AUROC (0.92) and AUPRC (0.51), followed by the logistic regression method (AUROC 0.90; AUPRC 0.59), light GBM (AUROC 0.89; AUPRC 0.52), and XGBoost (AUROC 0.86; AUPRC 0.45).

**Table 1:** Data characteristics of study population.

Characteristics			Total		FAS^a^ (n=20)		No FAS (n=575)
**Drinking timing, n (%)**							
	First trimester only	491		3 (0.6)		488 (99.4)	
	First and second trimesters	8		0 (0.0)		8 (100.0)	
	All throughout	95		17 (17.9)		78 (82.1)	
	Other	1		0 (0.0)		1 (100.0)	
**Race, n (%)**							
	American Indian or Alaska Native	79		1 (1.3)		78 (98.7)	
	Black	238		4 (1.7)		234 (98.3)	
	White	237		10 (4.2)		227 (95.8)	
	Other^b^	41		5 (12.2)		36 (87.8)	
**Ethnicity, n (%)**							
	Hispanic or Latino	92		2 (2.2)		90 (97.8)	
	Not Hispanic or Latino	503		18 (3.6)		485 (96.4)	
Maternal age at childbirth, mean (SD)			25.45 (3.59)		24.30 (2.93)		25.93 (3.61)
**Preferred alcoholic beverage, n (%)**							
	Beer	73		12 (16.4)		61 (83.6)	
	Wine	496		3 (0.6)		493 (99.4)	
	Liquor	26		5 (19.2)		21 (80.8)	
**Prenatal care, n (%)**							
	No	45		5 (11.1)		40 (88.9)	
	Yes	550		15 (2.7)		535 (97.3)	
**Pregnancy complications, n (%)**							
	No	468		16 (3.4)		452 (96.6)	
	Yes	127		4 (3.1)		123 (96.9)	
Total, n (%)			595		20 (3.4)		575 (96.6)

^a^FAS: fetal alcohol syndrome.

^b^Asian, Native Hawaiian or other Pacific Islander, or more than 1 race.

Shapley values illustrated that drinking throughout all 3 trimesters of pregnancy, maternal age, race, and type of alcoholic beverage consumed were observed to be the most important features for prediction (mean 1.08), followed by maternal age (mean 0.79), race (mean 0.52), beverage type (mean 0.4), pregnancy complications (mean 0.2), ethnicity (mean 0.07), and prenatal care (mean 0.03). Regarding alcohol consumption amount, drinking throughout all 3 trimesters was associated with FAS risk, however, only drinking during the first trimester, drinking during the first and second trimesters, and other patterns were not associated with increased FAS risk.

## Discussion

### Principal Findings

FAS is more common among socioeconomically disadvantaged populations but is also more likely to be underdiagnosed due to inadequate resources [34]. The significance of our research from an algorithm development perspective is that our study is the first of its kind to use ML algorithms to predict FASD onset, via the incorporation of variables associated with maternal pregnancy behaviors and the CIFASD data set. Despite FASDs being 100% preventable in nature, the application of automated methods such as ML for the identification of high-risk groups remains rare, relative to other neurodevelopmental disorders.

However, while few FASD studies have incorporated ML for “disease prediction” based on maternal behaviors and sociodemographic characteristics, a growing body of literature has been incorporating ML for “diagnostic purposes” using facial features’ data to distinguish FASD children from non-FASD children [35]. While this was not the objective of our study, it is interesting to note that recent studies amalgamating ML methods such as decision trees, support vector machine, and k-nearest neighbor with 3D-metric facial data for FAS diagnosis have been able to achieve an accuracy rate of up to 89% in clinical settings [35]. Scholars have remarked that advances in FASD diagnosis are often “hindered by a lack of consensus in diagnostic criteria and limited use of objective biomarkers,” highlighting the value of such studies to aid clinical decision-making.

For other intellectual disabilities with larger data sets like autism spectrum disorder (ASD) and epilepsy [36], accuracy rates have ranged from 72.40% [37] to 86.64% [38]. However, in 1 study of children with ASDs, data sets incorporating graph signal processing data were able to reach a diagnostic accuracy of up to 100% in differentiating ASD patients from typically developing children [39]. These studies suggest that for neurodevelopmental illnesses like ASD, artificial intelligence techniques could aid physicians to apply automatic diagnosis and rehabilitation procedures with great accuracy in the future [40].

In 2 of our ML models, the use of only 12 variables centralized around self-reportable measures of alcohol consumption during pregnancy and basic sociodemographic characteristics (age and race or ethnicity) resulted in a predictive accuracy of over 90%. While it may be common knowledge that drinking any amount of alcohol can harm the fetus, it is important to understand that information regarding dose, timing, type, and frequency can improve the prediction of FAS.

### Comparison to Prior Work

Regarding ML, scholars have emphasized that there are numerous issues with interpretability and inference, including overgeneralization or overinterpretation of causality [35]. Likewise, because our data set was small and FASD prevalence was low for numerous scenarios, certain confusion matrices had higher numbers for identifying true negatives than true positives, resulting in substantial imprecision in the estimates of “sensitivity” and “precision” [36]. While this may be common among rare outcomes, it highlights the need to gather more data on pregnant drinkers in future studies.

In our study, we also evaluated the AUPRC as a performance metric for FASD. Precision-recall curves are based on precision rather than the false-positive rate and are noted by scholars to be a better assessment of model performance when predicting outcomes that are rare or “unbalanced” due to a small data set [37]. While our curve was beneficial in helping understand the magnitude and uncertainty of each ML algorithm’s performance, in a real-world setting, skewed class distribution will likely be inevitable [38]. Thus, we recommend that instead of using ML algorithms alone, these models should be used in combination with other, externally validating screening or surveillance strategies to identify high-risk FASD groups, for example, women with a history of alcohol abuse during pregnancy or multiple children with FASDs [39].

### Strengths and Limitations

Our model only incorporates a small number of variables that could easily be collected by health systems via self-registrable questionnaires or routine data collection methods from health records [40]. This may be beneficial as some skepticism has been expressed regarding the feasibility of implementing ML models in real-life health care practices [41]. Among the different algorithms tested, the CatBoost algorithm had the highest predictive performance of all algorithms. As noted previously by other researchers, because CatBoost is the newest gradient-boosting decision tree algorithm with better handling of categorical features compared to other algorithms, it will usually outperform other models such as XGBoost and light GBM [42].

This study has several limitations. First, like all traditional studies of FASDs, some of the self-reported data on PAE is likely unreliable and influenced by social desirability or retrospective recall bias [43]. For example, it has been noted that pregnant women often present a more favorable image of themselves when it comes to self-reporting questionnaires about their dietary intake during pregnancy [44]. As a solution, researchers are increasingly exploring the use of biospecimens including meconium, urine, the placenta, neonatal blood, maternal blood, and fetal tissue (ie, the umbilical cord) to extract biomarkers like fatty acid ethyl esters, ethyl glucuronide, ethyl sulfate, and phosphatidylethanol to detect PAE [45]. Second, certain barriers to data collection were inevitable because of barriers such as a limited window of detectability, difficulties in collection, and high costs of analysis [45]. Such barriers may be overcome in future FASD studies if data on biomarkers are used in combination with maternal self-reporting to improve prediction. Third, besides logistic regression, our study mainly focused on boosting algorithms because they are known to reduce variance and have higher flexibility or interpretability in ML ensembles [46]. However, for training sets that are small like ours, boosting mechanisms may formulate discriminative classifiers where the optimality criterion that the loss function approximates is unclear [47]. Thus, future studies would benefit from incorporating other algorithms that are not boosting-based, such as random forest, for a more well-rounded analysis.

### Conclusions

In this study comparing multiple ML algorithms to predict FAS risk among a sample of pregnant drinkers, the CatBoost model outperformed both traditional and other ML models. The variables and methods used in our CatBoost model may serve as an effective, automated method for identifying high-risk groups in clinical predictions of FAS. Future research should evaluate the accuracy of such methods in predicting FAS relative to traditional approaches such as logistic regression analysis, as well as the extent to which certain risk factors may have been missed or overlooked, for overall improved clinical outcomes among FAS patients.

## Appendix

Multimedia Appendix 1 Box plot showing area under the receiver operating characteristic curve variation among 4 models. GBM: gradient-boosting machine.

## Abbreviations

ASD: autism spectrum disorder
AUD: alcohol use disorder
AUPRC: area under the precision-recall curve
AUROC: receiver operating characteristics curve
CIFASD: Collaborative Initiative on Fetal Alcohol Spectrum Disorder
FAS: fetal alcohol syndrome
FASD: fetal alcohol spectrum disorder
GBM: gradient-boosting machine
IRB: institutional review board
ML: machine learning
PAE: prenatal alcohol exposure

## Data Availability

Access to the National Institutes of Health’s Collaborative Initiative on Fetal Alcohol Spectrum Disorder (CIFASD) data set can be requested from [48]. The CIFASD Data Access Committee will review and make available archived data for all research that aims to improve diagnoses, interventions, and treatment of fetal alcohol syndrome disorders.
