# Supplementary figures and images for: Predicting Fetal Alcohol Spectrum Disorders Using Machine Learning Techniques: Multisite Retrospective Cohort Study
Source: J Med Internet Res. 2023 Jul 18;25:e45041. doi: 10.2196/45041 (PMC10394506; doi:10.2196/45041)

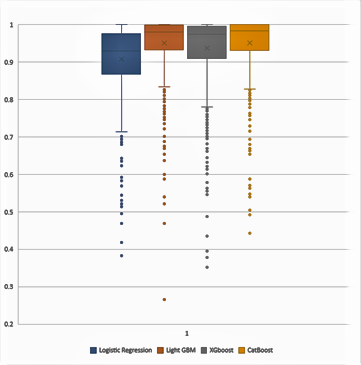

Supplement: Multimedia Appendix 1 [file jmir_v25i1e45041_app1.png]
